# Energy‐Based Device Management of Nodular Reaction Following Poly‐D, L‐Lactic Acid Injection for Tear Trough Rejuvenation

**DOI:** 10.1111/jocd.16575

**Published:** 2024-09-16

**Authors:** Suk Bae Seo, Jovian Wan, Kyu‐Ho Yi

**Affiliations:** ^1^ SeoAhSong Dermatologic Clinic Seoul Korea; ^2^ Asia‐Pacific Aesthetic Academy Hong Kong Hong Kong; ^3^ Division in Anatomy and Developmental Biology, Department of Oral Biology Human Identification Research Institute, BK21 FOUR Project, Yonsei University College of Dentistry Seoul Korea; ^4^ Maylin Clinic (Apgujeong) Seoul Korea

**Keywords:** adverse effect, dermal filler, nodule, polylactic acid, rejuvenation

## Abstract

**Background:**

Poly‐D,L‐lactic acid (PDLLA) is used for tear trough rejuvenation but can cause complications like nodular reactions. This report describes using a radiofrequency device to manage these nodules.

**Case Presentation:**

A 42‐year‐old woman developed firm, non‐inflammatory nodules 3 weeks after receiving PDLLA (Juvelook) injections in the tear trough area. The nodules were firm and not associated with erythema or tenderness.

**Intervention:**

The monopolar radiofrequency device was used directly on the nodules with 150 shots at an energy level 115 J, 28.75 J/cm². The treatment resulted in complete resolution of the nodules within 24 hours.

**Results:**

The radiofrequency treatment effectively resolved the nodular reaction without recurrence, highlighting the device's compatibility with the unique structure of Juvelook's PDLLA.

**Conclusion:**

Radiofrequency therapy is effective for managing nodular reactions following PDLLA injections. Further research is needed to optimise protocols and improve the safety of biostimulator treatments in cosmetic procedures.

## 1 Introduction

Dermal fillers such as hyaluronic acid (HA) have been widely employed in cosmetic procedures for decades. More recently, patients have increasingly opted for biostimulators over HA fillers, driven by a preference for the natural stimulation of collagen rather than the filling effect provided by HA products [1, 2, 3, 4, 5, 6]. Injectable poly‐lactic acid is recognized as a collagen‐stimulating filler. There are two variants: injectable poly‐L‐lactic acid (PLLA) and injectable poly‐D,L‐lactic acid (PDLLA), which are specifically used in this case report [5, 7, 8, 9, 10, 11]. Injectable PDLLA (Juvelook; VAIM Inc., Seoul, Korea) includes PDLLA and sodium hyaluronate as a carrier gel. It is supplied in vials as a lyophilized powder, necessitating reconstitution with a diluent before injection.

Tear trough deformity is characterized by a deepening of the crease beneath the lower eyelid, leading to a darkened and sunken appearance. There is a growing desire to address the cosmetic effects of aging, particularly in the eye area, resulting in an increase in the diagnosis and treatment of tear trough deformity [12, 13, 14], Treating the tear trough area is challenging because of the thin skin and racial variability of the region.

PDLLA induces collagen synthesis by stimulating fibroblasts and causing a subclinical inflammatory reaction. Although PDLLA is absorbable, biocompatible within human tissue, and has a relatively strong safety profile, complications such as nodules, papules, and granulomas have been documented in the literature [15, 16, 17, 18, 19].

There are reports of nodule formation as an adverse effect of using poly‐lactic acids, and there have not been established treatment plans for these side effects [16, 18, 19, 20]. Previously, these nodules were treated with excisions [20]. This case study presents a case of an unusual nodular reaction following a PDLLA injection for the management of tear trough deformity and its subsequent treatment.

## 2 Clinical Case

**FIGURE 1:**
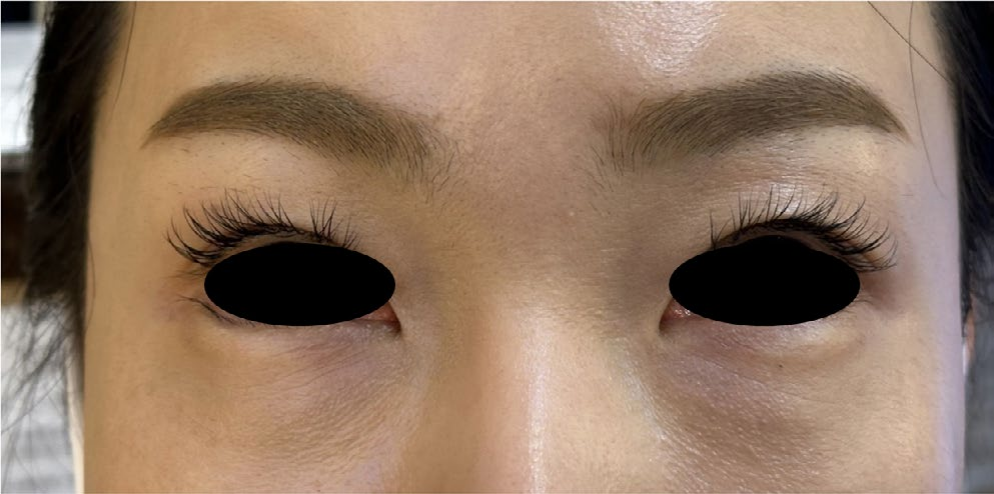
Prior photograph of a 42‐year‐old immunocompetent woman showing tear trough hollows secondary to aging. The patient received bilateral poly‐D, L‐lactic acid injections in the tear trough region.

A 42‐year‐old immunocompetent woman presented with tear trough hollows secondary to aging and received PDLLA injections bilaterally in the tear trough region (Figure 1). Prior to this treatment, she had received three PDLLA injections to her forehead and temples over a year ago but had no previous aesthetic treatments to the tear trough region. Concurrently with the PDLLA injections for her tear troughs, she also received botulinum neurotoxin type A injections for the glabellar region and lateral canthal rhytids. Her medical history was unremarkable, with no known allergies.

The procedure involved the use of PDLLA with a larger particle size, which led to adverse effects. This case occurred when the procedure was performed by a practitioner who was conducting it for the first time. In this case, the injectable PDLLA used was Juvelook Volume (Lenisna, VAIM Inc., Seoul, Korea). Each vial contains 200 mg of the product, comprising 170 mg of PDLLA and 30 mg of HA, with a particle size ranging from 40 to 60 μm. On the day of the procedure, one vial was reconstituted with 8 mL of normal saline and vortexed for 30 min before being used for tear trough rejuvenation. The patient received one session where 2 mL of PDLLA was injected into each tear trough. There was no immediate response to the injections posttreatment.

**FIGURE 2:**
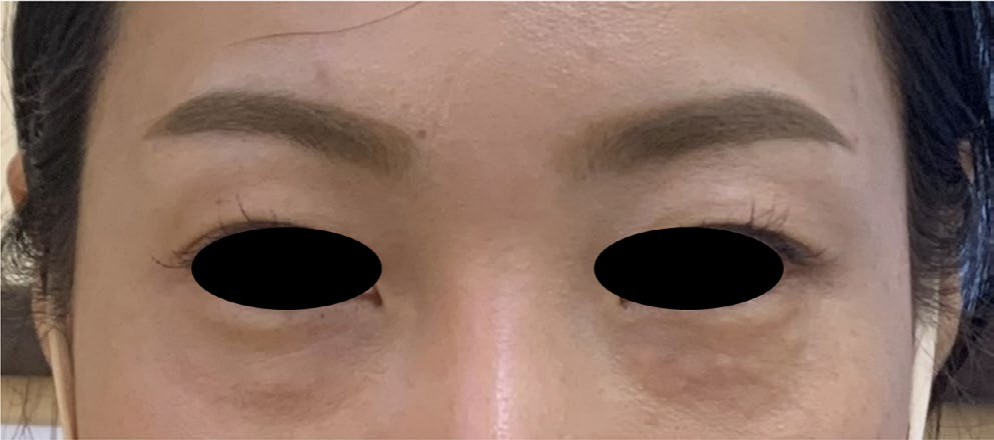
Photograph 3 weeks posttreatment, showing small, noninflammatory nodules in both tear trough regions. Nodules are firm and hardened, without erythema, tenderness, fluctuance, or ulceration, surrounded by minor soft tissue swelling.

Three weeks after the treatment, the patient presented with visible, small, noninflammatory nodules in both tear trough regions (Figure 2). These nodules were firm and hardened but exhibited no signs of erythema, tenderness, fluctuance, or ulceration. The nodules were surrounded by minor soft tissue swelling. The patient had no fever, and a comprehensive systemic review revealed no other abnormalities. To date, the nodules have not recurred.

The nodule occurred because the practitioner used the wrong product. Juvelook (VAIM Inc., Seoul, Korea), which has a particle size of 20 to 30 μm, typically does not cause nodules. However, the practitioner used Juvelook Volume, which has a larger particle size of 40–60 μm.

**FIGURE 3:**
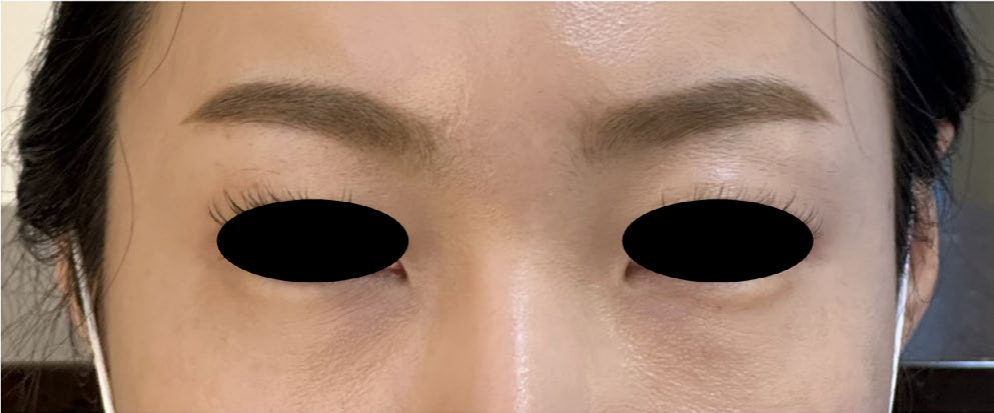
Photograph taken 24 h post‐monopolar radiofrequency treatment, demonstrating complete resolution of nodules after a single session.

The monopolar radiofrequency device was used directly on the tear trough area with 150 shots administered using 4 cm^2^ tips. The maximum energy level was set to 115 J or 28.75 J/cm^2^. After two sessions, the nodules completely resolved within 24 h (Figure 3). [Correction added on 27 September 2024, after first online publication: In the preceding sentence, “one session” has been changed to “two sessions” in this version.] Typically, patients begin treatment at the highest energy level of 5. If they experience discomfort due to the heat, the intensity is reduced to 95 J, 23.75 J/cm^2^, and if necessary, further reduced to 75 J, 18.75 J/cm^2^. Strong compression was applied following the use of a monopolar radiofrequency device.

## 3 Discussion

In this study, we were the first to use energy‐based devices for the thermal degradation of PLA‐induced nodules. Specifically, we employed monopolar radiofrequency (1–2 MHz) treatment, using a thermometer to measure the surface temperature, which was maintained at 41–42°C [21]. The treatment involved either contact‐type or rolling‐type radiofrequency application. The mechanism for resolving the nodules is based on the concept of glass transition temperature (the temperature at which a material becomes malleable, like glass when heated). For PLLA, this temperature is around 60°C. However, for PDLLA, which is porous and hydrated, the glass transition can occur at 38–39°C, potentially explaining the treatment's effectiveness. The authors believe this approach is applicable specifically to PDLLA. Additionally, previous literature suggests that using multifrequency radiofrequency devices can induce M2 polarization, which may further support the effectiveness of this treatment method [22].

Hybrid fillers, such as calcium hydroxyapatite (CaHA) combined with HA or poly‐L‐lactic acid (PLA) combined with HA, have recently gained popularity because of their various advantages [23, 24, 25]. In the tear trough region, aging often leads to the reduction in fat in the superficial layer of the orbicularis oculi muscle, making the area appear more pronounced [26, 27]. Injecting HA filler superficially in this region can easily cause the Tyndall effect. To prevent this, PLA can be injected superficially, whereas HA filler can be used in deeper layers [28]. However, injecting PLA superficially can lead to the formation of nodules. A solution to this issue is the use of energy‐based devices for the thermal degradation of the nodules.

Although nodules and granulomas are uncommon adverse events following treatment with biostimulators, they can cause significant cosmetic and psychological concerns for patients [26, 29]. The literature indicates that persistent, intractable nodules can remain despite multiple treatments, such as hyaluronidase and systemic or intralesional steroids. Surgical excision, typically considered a last resort, poses risks of complications, including incomplete removal of the granuloma and scar formation [30, 31].

It is crucial to differentiate between granuloma and nodular reactions because of their distinct aetiologies and clinical manifestations. Granulomas primarily arise from an exaggerated inflammatory response of the host and are characterized by significant edema and a substantial, hard granulomatous reaction that is both palpable and visible at the injection sites. They typically manifest between 6 and 24 months postinjection and can attain considerable size. Treatment with intralesional steroids is recognized as effective for managing granulomas. The diagnosis of granuloma should be confirmed by histopathological evaluation [32, 33, 34].

Conversely, nodules primarily result from improper product deposition or accumulation within dynamic facial musculature. Clinically, nodules present as discrete, palpable lesions that may vary in visibility depending on their anatomical location, such as the neck, hands, or forehead. They typically appear within 1–2 months following the procedure and are usually solitary, ranging in size from small peas to larger lentils. Although some nodules respond favorably to intralesional steroid therapy, surgical intervention may be warranted in severe cases [15, 17, 20, 35, 36]. In our case, the clinical presentation strongly suggests nodular reactions, and the patient had no prior medical history or identifiable event that could plausibly trigger such a reaction.

**FIGURE 4:**
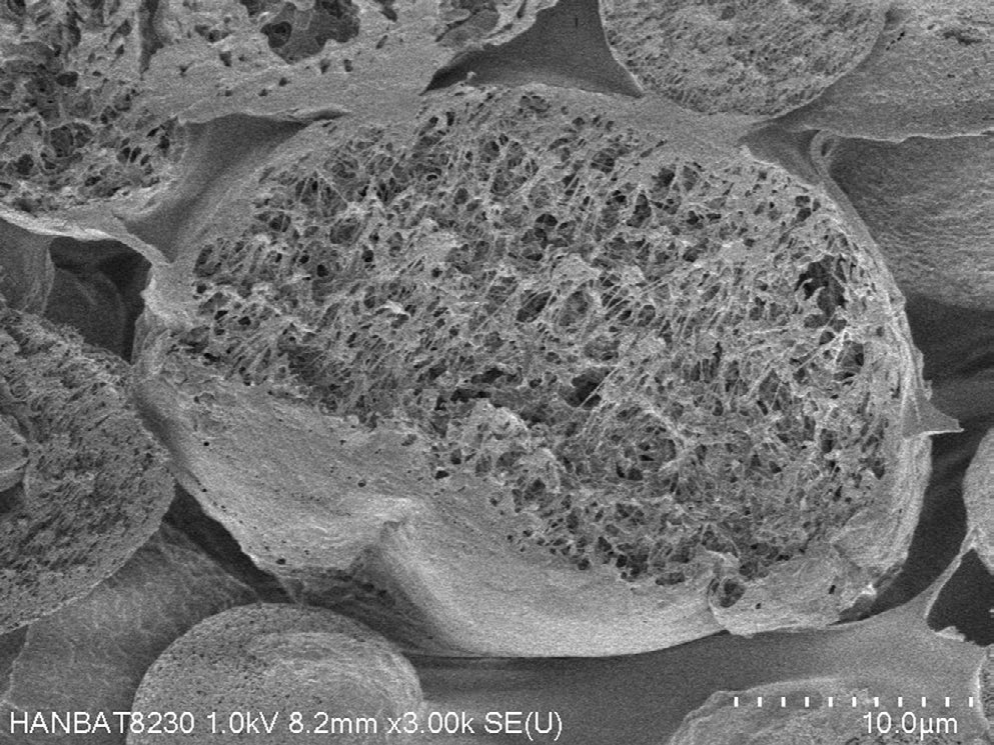
Scanning electron microscopy of Juvelook (VAIM Inc., Seoul, Korea) particle structure. Juvelook (VAIM Inc., Seoul, Korea) features a hybrid filler combining hyaluronic acid's immediate volumizing effects with poly‐D, L‐lactic acid's long‐term collagen stimulation. The poly‐D, L‐lactic acid particles exhibit a distinctive porous and reticular inner structure within an outer spherical and foamy shape. The authors hypothesize that this porous design facilitates gradual breakdown with radiofrequency.

Juvelook (VAIM Inc., Seoul, Korea) is a hybrid filler that merges the immediate volumizing effects of HA with the long‐term collagen‐stimulating benefits of PDLLA. The PDLLA in Juvelook has a distinctive structure, featuring an outer spherical and foamy shape combined with an inner reticular and porous design. This patented structure allows for gradual decomposition from the inside, leading to a slow and slight change in acidity around the particles, which improves tissue compatibility (Figure 4).

Based on our understanding, the porous nature of Juvelook's PDLLA allows for more effective degradation when energy‐based devices are applied. There have been reports of refractory nodules developing after filler treatments that have been successfully resolved using a radiofrequency device [31, 37].

In conclusion, our case highlights the successful management of nodular reactions following PDLLA injections for tear trough deformity in a healthy 42‐year‐old woman using a monopolar radiofrequency device.

## Author Contributions

Conceptualization and visualization: Suk Bae Seo and Jovian Wan. Writing – original draft preparation: Jovian Wan, Suk Bae Seo, and Kyu‐Ho Yi. Writing – review and editing: Jovian Wan. Supervision: Kyu‐Ho Yi. All authors have reviewed and approved the article for submission.

## Conflicts of Interest

I acknowledge that I have considered the conflict of interest statement included in the “Author Guidelines.” I hereby certify that to the best of my knowledge, no aspect of my current personal or professional situation might reasonably be expected to significantly affect my views on the subject I am presenting.

## Data Availability

Data sharing is not applicable to this article as no new data were created or analyzed in this study.
